# The Effectiveness of Oral Vancomycin on Inflammatory Bowel Disease in Patients With Primary Sclerosing Cholangitis: A Systematic Review

**DOI:** 10.1093/ibd/izae257

**Published:** 2024-11-04

**Authors:** Naik Arbabzada, Liz Dennett, Guanmin Meng, Farhad Peerani

**Affiliations:** Division of Gastroenterology, Department of Medicine, University of Alberta, Edmonton, AB, Canada; Division of Gastroenterology, Department of Medicine, University of Alberta, Edmonton, AB, Canada; Geoffrey and Robyn Sperber Health Sciences Library, University of Alberta, Edmonton, AB, Canada; Division of Gastroenterology, Department of Medicine, University of Alberta, Edmonton, AB, Canada; Division of Gastroenterology, Department of Medicine, University of Alberta, Edmonton, AB, Canada

**Keywords:** primary sclerosing cholangitis, inflammatory bowel disease, oral vancomycin

## Abstract

**Background:**

Approximately 70% of primary sclerosing cholangitis (PSC) patients have inflammatory bowel disease (IBD). The IBD therapies currently used to treat PSC-IBD patients have side effects and can be costly. Oral vancomycin (OV)—a safe, economical, and convenient therapy—has been reported to be a salvage therapy in refractory PSC-IBD patients. This systematic review aims to summarize the current literature regarding the effectiveness and safety of OV to treat IBD in PSC patients.

**Methods:**

A systematic literature review of Scopus, Embase, Web of Science, MEDLINE, and CINAHL was performed until March 2024. The Murad scale, Newcastle-Ottawa scale, and Cochrane Collaboration Risk of Bias Tool were used to determine the quality of the case reports and case series, cohort studies, and randomized controlled trial (RCT), respectively. The outcomes sought were response or remission across clinical, biochemical, endoscopic, and histological parameters.

**Results:**

Of the 1725 published studies, we identified 9 case reports, 7 case series, 3 cohort studies, and 1 RCT. Most studies reported an improvement in clinical IBD symptoms such as diarrhea and hematochezia. Fewer publications provided supporting objective data in the form of fecal calprotectin, endoscopic Mayo scores, and histology. There were no reports of vancomycin-resistant enterococci infections.

**Conclusions:**

Oral vancomycin appears safe and effective to treat IBD in a subset of PSC patients. Future studies would benefit from prospective data collection incorporating standardized symptomatic, endoscopic, and histologic indices. Ultimately, a well-powered RCT is needed to better assess the effectiveness, safety, and durability of OV therapy.

**Key Messages**

**What is already known?**

Oral vancomycin (OV) is a safe non-absorbable antibiotic that may be effective in treating inflammatory bowel disease (IBD) in primary sclerosing cholangitis (PSC) patients who can be refractory to small molecule or biologic therapy.

**What is new here?**

The current systematic review summarizes the clinical data supporting the use of OV to treat IBD in PSC patients and identifies gaps in knowledge.

**How can this study help patient care?**

This systematic review helps care providers contextualize the available data and paves the way to guide future clinical trials in the area to ultimately help patients with this orphan disease.

## Introduction

Primary sclerosing cholangitis (PSC) is a chronic cholestatic liver disease characterized by biliary inflammation and fibrosis of the intrahepatic and extrahepatic bile ducts.^1^ In North America and Western Europe, the incidence of PSC ranges from 1 to 1.5 cases per 100 000 person-years, while the prevalence ranges from 6 to 16 cases per 100 000 person-years.^2^ Unfortunately, there is no effective treatment for PSC with many patients requiring liver transplantation due to end-stage liver disease. Uniquely, approximately 70% of PSC patients also have co-existing inflammatory bowel disease (IBD)—with ulcerative colitis (UC) being the most common phenotype.^3^

Although the majority of PSC patients with IBD have the diagnosis of UC, PSC patients with IBD often exhibit a phenotype that is uniquely distinct from classic Crohn’s disease (CD) and UC. The PSC-IBD phenotype is characterized by a predominantly asymmetric colonic disease distribution (right > left), that is, non-stricturing/non-penetrating with rectal sparing and backwash ileitis, varied microbiota composition, and often a quiescent disease course.^3,4^ The clinical course is influenced by the bidirectional relationship of the gut-liver axis.^3,5^ Although IBD symptoms are generally mild, endoscopic, and histologic disease activity often persists^4^ placing PSC-IBD patients at an increased risk of colorectal dysplasia and neoplasia.^3,6^ Furthermore, some patients post-liver transplant can be refractory to conventional IBD therapies necessitating surgical management.^7–12^ Resorting to colectomy has unique implications in the PSC-IBD patient including the risk of parastomal varices and the morbidity from major intra-abdominal surgery depending on the status of a patient’s liver disease.^13^ Lastly, in an era where small molecule and biological therapies are more frequently prescribed to treat IBD, the accumulating costs to society as well as the adverse effects of immunosuppression need to be taken into consideration.

Although the etiology and pathogenesis of PSC-IBD are unknown, distinct gut microbiome profiles suggest that the microbiome may play a significant role and be a potential therapeutic target for PSC-IBD.^14–17^ When compared to healthy controls and IBD patients, patients with PSC-IBD exhibit more severe dysbiosis, characterized by lower microbiota diversity and greater abundance of specific taxa, including *Barnesiellaceae*, *Blautia*, *Enterococcus*, *Escherichia*, *Fusobacterium*, *Lactobacillus*, *Lachnospiraceae*, *Megasphaera*, *Parvimonas* sp., *Staphylococcus*, and *Veillonella*,^15–20^ as well as a reduction in *Faecalibacterium*, Bacteroides, Enterobacteriaceae, Clostridiales II, Gammaproteobacteria, Lentisphaerae, Myxococcales, Prevotella, Paraprevotellaceae, and *Roseburia.*^17–19^ Manipulating the gut microbiome is an old concept in IBD^21^ resulting in the use of antibiotics^22–24^ and more recently fecal microbial transplantation.^25^ Oral vancomycin (OV), a non-absorbable glycopeptide antibiotic, has a narrow spectrum of antimicrobial activity. It also functions as a gut immunomodulator by inhibiting the release of cytokines from T cells^26^ or reducing the release of nuclear factor kappa B-dependent proinflammatory cytokines like tumor necrosis factor-alpha (TNF-α) and interleukin-1 beta.^27^ Moreover, OV has been demonstrated to inhibit the production of carcinogenic secondary bile acids by altering the gut microbiome.^28^

As both PSC and IBD are associated with abnormal immune responses and changes to the intestinal microbiome, OV has been used as salvage IBD therapy in refractory PSC-IBD patients in multiple centers across the world with encouraging results.^8,9,12,29–33^ A systematic literature review was conducted to summarize the current knowledge of the effectiveness of OV in the treatment of IBD in PSC patients, which will help inform further research in the field of managing the PSC-IBD phenotype.

## Methods

### Literature Search

**Figure 1.**
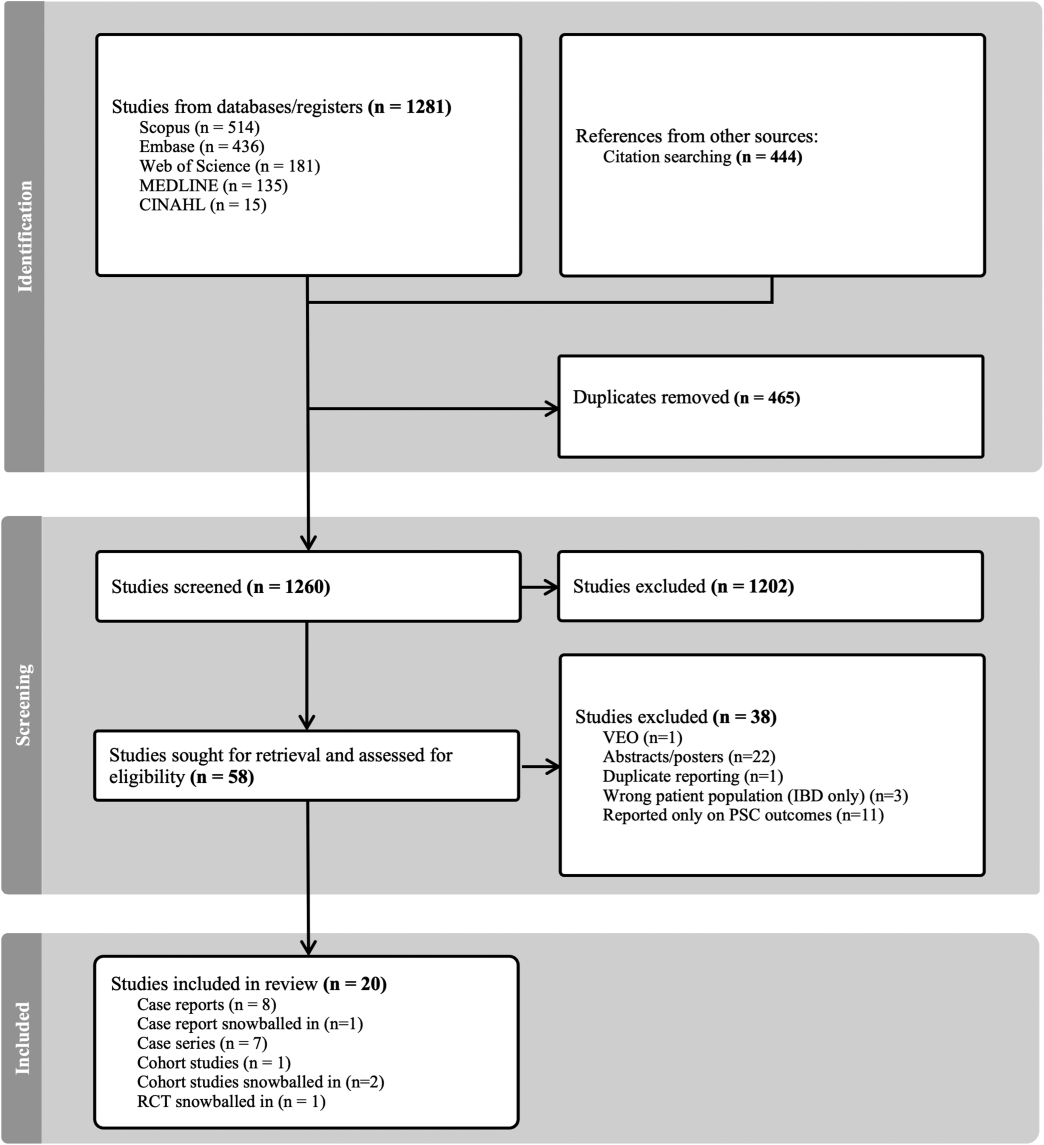
Flow diagram of study selection. IBD, inflammatory bowel disease; PSC, primary sclerosing cholangitis; RCT, randomized control trial; VEO, very early-onset inflammatory bowel disease.

A health sciences librarian (L.D.) conducted searches in Ovid Medline(R) ALL, Embase (Ovid Interface), CINAHL Plus with Full Text (EBSCO host interface), Scopus, and Web of Science Core Collection (including Science Citation Index Expanded, Social Sciences Citation Index, Arts & Humanities Citation Index, Emerging Sources Citation Index, Conference Proceedings Citation Index—Science, Conference Proceedings Citation Index—Social Science & Humanities) from database inception until March 22, 2024. The search combined subject headings and free-text terms for 3 concepts: inflammatory bowel disease, PSC, and antibiotics. The search strategy was optimized for each database and no date, language, publication, or study design filters were applied. Reference lists of included articles and reviews were reviewed for additional studies. A flowchart demonstrating the manuscript selection process is provided in Figure 1. The full details of the search strategy can be found in Supplementary Materials.

Two reviewers (G.M. and N.A.) independently screened the title and abstract of studies identified by the search. The full texts of published manuscripts were then assessed to ensure they met inclusion criteria. Any disagreement was resolved by consensus with a third reviewer (F.P.).

### Selection Criteria

Inclusion criteria were as follows: (1) peer-reviewed case reports, case series, research letters, or case-controlled, retrospective, prospective, or randomized controlled studies including PSC-IBD patients published in full manuscript form, and (2) articles that assessed the effectiveness or efficacy of OV on clinical, biochemical (fecal calprotectin or FCP), endoscopic, or histologic IBD outcomes either as a primary or secondary outcome. Studies were excluded that could not be retrieved in English, that focused on very early-onset IBD and that focused on the treatment of pouchitis in patients with PSC-IBD post colectomy.

### Quality Assessment

A case report was defined as a report on 1 patient whereas a case series was defined as summarizing the clinical course of 2 or more patients. The quality of the case series and case reports was assessed using the 8-point scale as outlined by Murad and colleagues.^34^ The questions deemed critical were ascertainment, causality, and reporting since PSC-IBD is a rare disease. Furthermore, the randomized controlled trial (RCT) was assessed using the Cochrane Collaboration Risk of Bias Tool^35^ and the cohort studies were assessed based on the Newcastle-Ottawa scale.^36^

## Results

The initial database search yielded 1281 abstracts and citation searching yielded 444 abstracts. A total of 58 studies were reviewed at the full-text stage, of which 20 were eligible for inclusion. We identified 8 case reports, 7 case series, and 1 cohort study that met the eligibility criteria through our systematic review with 1 RCT, 2 cohort studies, and 1 additional case report that were snowballed in from citation searching (Figure 1).^1,37–39^ Notably, none of the studies commented on the presence of adverse drug reactions while on OV therapy. The average quality of the case reports and case studies was 5.6 out of 8 (Supplementary Table 1). The RCT had an overall low risk of bias on the Cochrane Collaboration Risk of Bias Tool (Supplementary Table 2). Two of the cohort studies^1,40^ were of good quality (3 or 4 stars in the selection domain AND 1 or 2 stars in the comparability domain AND 2 or 3 stars in the outcome/exposure domain) and one of the cohort studies^39^ was poor quality (Supplementary Table 3). Regarding study selection, there were no disagreements between the primary reviewers G.M. and N.A.

### Case Reports

There were 9 case reports (4 pediatric and 5 adult) describing refractory PSC-UC patients (6 out of 9 failed or were intolerant to at least 2 biologics). Patient ages ranged from 9 to 51 years with OV dosing being as low as 125 mg PO BID and as high as 1000 mg PO TID (Table 1). Clinical improvement was reported in 8 out of 9 cases with the most rapid response occurring within 2 weeks and 2 studies reporting symptomatic immediate improvement within 24 hours^9,41^; however, the patient described by Dubrovsky and Kitts^37^ demonstrated no change in gastrointestinal symptoms. Biochemical improvement in the form of FCP was described in 6 out of 9 patients.^9,30,31,33,42,43^ Endoscopic improvement as measured by the Mayo score was demonstrated in 5 out of 9 patients^8,9,30,33,44^ while histologically quiescent disease was commented on in 4 out of 9 patients.^9,33,43,44^

**Table 1.** Summary of case reports on the effect of OV in the management of IBD in PSC patients.

Author/date	Population	Therapy	Outcomes				
				Clinical	Biochemical	Endoscopic	Histologic
Rahman et al., 2021^8^	1 [51M PSC-UC, OLTx]	OV 125 mg BID + infliximab 10 mg/kg q 4 weeks + MMF 1 g BID + tacrolimus 1 mg BID; infliximab was then reduced to 5 mg/kg q 8 weeks after 10 months of OV Duration: 10 months	Pre-OV	Bloody diarrhea		Mayo 3 pancolitis	Active inflammation with crypt distortion and crypt abscesses
			With OV	Corticosteroid-free clinical remission [3 months]		Mayo score 1 [3 months]	
Ahmed et al., 2023^9^	1 [30F PSC-UC, OLTx]	OV 125 mg QID + tacrolimus Duration: Initial 28 days, then re-treated for 3 months	Pre-OV	Urgency, frequency, and hematochezia	FCP 492 µg/g	Mayo 3 pancolitis	
			With OV	Decreased stool frequency, resolution of urgency, and hematochezia [immediate response both times]	FCP 43 µg/g [6 weeks]	Mayo score 0 [3 months]	Inactive chronic colitis
Dubrovsky and Kitts 2018^37^	1 [20F PSC-UC]	OV 500 mg TID →750mg TID →1000 mg TID Duration: Not reported	Pre-OV	Bloody diarrhea, frequency, abdominal pain			
			With OV	No change			
Perez Martin et al., 2023^31^	1 [13 PSC-UC]	OV 375 mg Q6H (50 mg/kg/d) + ustekinumab Duration: 10 months	Pre-OV	8 bloody liquid stools per day	FCP >2000 µg/g		
			With OV	2 formed stools per day [18 days]	FCP 262 µg/g [10 months]		
Britto et al., 2021^42^	1 [9M PSC-UC]	OV 125 mg QID Duration: 246 days	Pre-OV	PUCAI 25	FCP 2000 µg/g	Mild pancolitis	
			With OV	PUCAI 5 [3 months]	FCP normalized [70 days]		
Almomen et al., 2023^33^	1 [34M PSC-UC, OLTx]	OV 500 mg BID + ustekinumab 90 mg q 4 weeks + tacrolimus 1 mg BID; OV reduced to 250 mg BID 6 months later Duration: 6 months	Pre-OV	Not reported	FCP 1600 µg/g	Mayo 3 pancolitis	
			With OV	Clinical remission [3 months]	FCP 277 µg/g [6 months]	Mayo score 0 [6 months]	Quiescent colitis
Kinderlehrer 2020^43^	1 [9M PSC-UC]	OV 500 mg TID + mesalamine 400 mg TID Duration: Not reported	Pre-OV	Diarrhea, abdominal pain	FCP 188 mg/kg	Left-sided Mayo 2 colitis	Mild active inflammation with mild architectural changes
			Pre-OV	Clinical remission [1 month]	Normalized FCP		
Nguyen et al., 2024^30^	1 [25F PSC-UC]	OV 500 mg BID + tacrolimus + ustekinumab induction (single 6 mg/kg dose). Tacrolimus was discontinued after 9 months and maintained on 90 mg SC q 8-weekly ustekinumab OV was weaned down to 125 mg daily after 2 attempts at discontinuation Duration: 18 months	Pre-OV	Diarrhea and abdominal pain	FCP 3500 µg/g	Deep ulceration of the anal canal and serpiginous ulceration of the rectosigmoid colon	
			With OV	Clinical remission [6 weeks]	FCP 312 µg/g [2 months]	Inactive disease [3 months]	
Buness et al., 2021^44^	1 [15F PSC-UC]	OV 500 mg TID (35 mg/kg/d); varied doses up to 1000 mg BID and various brands were used Duration: 10 yrs	Pre-OV	Diarrhea		Moderate chronic active UC	
			With OV	Formed stool [within 2 weeks]		Normal	Quiescent to mild chronic colitis

Abbreviations: FCP, fecal calprotectin; MMF, Mycophenolate mofetil; OLTx, orthotic liver transplant; OV, oral vancomycin; PSC, primary sclerosing cholangitis; PUCAI, pediatric ulcerative colitis activity index; SC, subcutaneous; UC, ulcerative colitis; yrs, years.

### Case Series

We extracted 7 case series (4 pediatric and 3 adult) from our literature review that described 65 PSC-IBD patients. Patient ages ranged from 2 to 69 years and OV doses ranged between 250 mg and 1.5 g PO daily (Table 2). In the 5 studies that captured previous IBD medication history, 7 patients were refractory or intolerant to 1 biologic and another 7 were refractory or intolerant to 2 biologics.^12,29,32,41,45^ The majority of case series reported on clinical IBD symptoms, and all demonstrated an overall improvement with OV with one patient reportedly demonstrating a clinical response within 24 hours.^41^ Biochemical remission was reported in 2 case series^32,45^ and endoscopic improvement, described as early as 5 months, was documented in 4 case series^12,29,32,45^ accompanied by some supporting histological changes.

**Table 2.** Summary of case series on the effect of OV in the management of IBD in PSC patients.

Author and date	Population	Therapy	Outcomes				
				Clinical	Biochemical	Endoscopic	Histologic
Dao et al., 2019^12^	8 [19-52 yrs; PSC-UC; 2-M, 6-F; 6 OLTx]	OV 125 mg PO QID × 6-8 weeks then tapered to 125 mg PO BID or TID Duration: 9-36 months	Pre-OV	Mean partial Mayo score 5.8		Mayo score 2	
			With OV	Mean partial Mayo score 0.4 [within 6-12 months]		Mayo score 0 (*n* = 6), 1 (*n* = 1) [within 6-12 months]	
Abarbanel et al., 2013^a^^26^	14 [2-18 yrs; 13-UC, 1-CD; 12-M, 2-F]	OV 50 mg/kg/d TID Duration: 12 months	Pre-OV	Abdominal pain, diarrhea			Acute and chronic colitis, cryptitis, lymphoplasmacytic infiltrate (*n* = 9)
			With OV	Clinical remission [within 2 weeks]			Normal or minimal inflammation (*n* = 9)
Tan et al., 2019^32^	17 [4-17 yrs; PSC-UC; 11-M, 6-F; 4 OLTx]	No dose provided Duration: Mean of 8.1 months	Pre-OV	PUCAI 26 (range 0-60)	FCP 1055 (*n* = 15)	Mayo score 1 (*n* = 4), 2 (*n* = 7), 3 (*n* = 6) pancolitis	
			With OV	PUCAI 1.8 (range 0-20)	FCP ≤150 (*n* = 15)	Mayo score 0 (*n* = 15)	Normal histology (9/15)
de Chambrun et al., 2018^29^	3 [20F, 69M, 24M; PSC-UC]	OV 500 mg BID Duration: 20.9-49.2 months	Pre-OV	PGA 2-3		Mayo 2-3 pancolitis	
			With OV	PGA 0		Mayo score 0 [5 months (*n* = 1), 6 months (*n* = 2)] [maintained response at 3 yrs in *n* = 1]	No histological inflammation (*n* = 3)
Shah et al., 2022^45^	6 [22-53 yrs; PSC-UC; 5-M, 1-F; 3 OLTx]	OV 250 mg to 1.5 g/d Duration: 9-31 months	Pre-OV	Mean partial Mayo score 7.3	Mean FCP 873 µg/g	Mayo 2-3 pancolitis	
			With OV	Mean partial Mayo score 0	Mean FCP 43 µg/g	Mayo score 0 (*n* = 3), 1 (*n* = 2)	No histological inflammation (*n* = 3)
Cox and Cox, 1998^41^	3 [15M, 14F, 14M; 1-UC, 2-CD]	OV 125 mg TID, OV 250 mg BID, and TID episodically Duration: 6-14 months	Pre-OV	Bloody diarrhea, abdominal pain			Acute and chronic colitis
			With OV	Clinical remission (2/3) [response within 2 weeks reported in *n* = 1, in *n* = 1 where OV was restarted, symptoms resolved within 24 h]			
Davies et al., 2008^a^^46^	14 [2-17 yrs; 11-UC, 3-CD; PSC-UC, 2.3:1 M:F]	OV 50 mg/kg/d Duration: 30-118 months	Pre-OV	Diffuse abdominal pain [*n* = 1]			Duodenal chronic active inflammation with granuloma (*n* = 1)
			With OV	Resolved [in 3 days]			Normal duodenum (*n* = 1)

^a^Some patients between these 2 studies were duplicates.

Abbreviations: CD, Crohn’s disease; FCP, fecal calprotectin; OLTx, orthotic liver transplant; OV, oral vancomycin; PGA, physician global assessment; PSC, primary sclerosing cholangitis; PUCAI, pediatric ulcerative colitis activity index; UC, ulcerative colitis; yrs, years.

### Cohort Studies and RCT

There were 3 cohort studies and 1 RCT that reported on a total of 422 mixed pediatric and adult PSC-IBD patients treated with OV. Rahimpour et al^38^ reported on a randomized placebo-controlled clinical trial, Ali et al^39^ was an open-label prospective cohort study, Deneau et al^1^ was a retrospective propensity-matched cohort study, and Ricciuto et al^40^ was a retrospective matched cohort study. Approximately 28% and 23% of PSC-IBD patients treated with OV in the studies by Deneau et al and Ricciuto et al, respectively, were on biologics, while biologic-exposed patients were excluded from Rahimpour et al and Ali et al’s studies. Patient ages ranged from 1.5 to 65 years and OV doses ranged between 500 and 2250 mg daily (Table 3). In 3 studies, clinical IBD symptoms were captured as a secondary outcome with the RCT and 1 cohort study both reporting an improvement in diarrhea.^1,38,39^ In Deneau et al, all 3 treatment groups (OV, ursodeoxycholic acid, no therapy) demonstrated an increased proportion of patients with a physician global assessment (PGA) of 0 at follow-up and moreover, colectomy rates were similar amongst the groups (5% vs 6% vs 9%, *P* = .629).^1^ At 1 year, Ricciuto et al^40^ reported an increased odds of clinical remission based on PGA (adjusted odds ratio [aOR] 5.24, 95% CI 2.68-10.22) and endoscopic remission (aOR 2.76, 95% CI 1.002-7.62).

**Table 3.** Summary of cohort studies and clinical trial on the effect of OV in the management of IBD in PSC patients.

Author/date/type	Population	Therapy	Clinical outcomes	
Deneau et al., 2021^1^ Retrospective propensity-matched cohort study	264 [7.9 to 15.6 yrs; 86% with IBD]	OV: 50 mg/kg/d if <30 kg or 1500 mg daily if ≥30 kg (*n* = 88)	Pre-OV With OV	PGA 0 (38%) PGA 0 (77%)
		UDCA: median 15 mg/kg/d (*n* = 88)	Pre-UDCA With UDCA	PGA 0 (2%) PGA 0 (47%)
		No therapy (*n* = 88) Duration: 1 yr	Baseline Follow-up at 1 yr	PGA 0 (6%) PGA 0 (27%)
Ali et al., 2020^39^ Prospective open-label observational cohort study	59 [1.5-44 yrs; 51-UC, 5-CD; 38-M, 21-F] *22 pediatric patients were also included in previous publications (Abarbanel et al., 2013 and Davies et al., 2008)	50 mg/kg/d TID if <30 kg or 500 mg TID if ≥30 kg (range 500-2250 mg daily) 17 patients stopped and restarted OV after 3 months, 12 patients discontinued OV after 3 months Duration: Minimum of 3 months, median of 2.7 yrs	Pre-OV	IBD-related diarrhea (43/56)
			With OV	Improvement in diarrhea (41/43) [median time 3 months]
Ricciuto et al., 2024^40^ Retrospective multicenter cohort study	70 pediatric [47-M, 23-F] OV patients were matched to 210 control patients based on IBD duration at the time of PGA assessment	Median OV dose of 16 mg/kg/day for a median of 2.5 yrs	OV was associated with greater odds of IBD clinical remission based on PGA (aOR 5.24, 95% CI 2.68-10.22) and endoscopic remission (aOR 2.76, 95% CI 1.002-7.62) [Outcome measured at 1 yr]	
Rahimpour et al., 2016^38^ Triple-blinded, randomized, placebo-controlled clinical trial	29 [19-65 yrs; 17-M, 12-F; 21/29 had IBD]	Placebo (*n* = 11) vs OV 125 mg QID (*n* = 18) × 12 weeks	Abdominal pain (*P* = .36) Diarrhea (*P* = .018) Blood in stool (*P* = .36) [within 1 month]	

Abbreviations: aOR, adjusted odds ratio; CD, Crohn’s disease; FCP, fecal calprotectin; IBD, inflammatory bowel disease; OV, oral vancomycin; PGA, physician global assessment; PSC, primary sclerosing cholangitis; UC, ulcerative colitis; UDCA, ursodeoxycholic acid; yrs, years.

## Discussion

In this systematic review, we summarized the findings of 9 case reports, 7 case series, 3 cohort studies, and 1 RCT. Acknowledging the limitation of publication bias favoring positive results, the most consistent result reported across all studies was an improvement in clinical IBD symptoms. The case reports and case series provided data supporting improvements in objective disease parameters including FCP, endoscopy, and histology. It is encouraging that Tan et al^32^ reported that 15 of 17 patients on OV had an endoscopic Mayo score of 0 and all patients had an FCP ≤150. Further support for the use of OV to treat PSC-IBD was highlighted by the improvement in symptoms after patients were re-challenged with OV.^9,26,30,41,46^ The duration of clinical remission of OV ranged from 2 weeks to 9 months in these studies, suggesting that ongoing maintenance therapy is needed. Interestingly, Buness et al^44^ reported on the differential efficacy of generic vancomycin capsules vs the originator Vancocin in a PSC-IBD patient potentially explained by the varied bioavailability of the different formulations and brands. The most compelling data was reported by Ricciuto et al^40^ which demonstrated an increased odds of clinical and endoscopic remission at 1 year in pediatric patients wherein clinical remission was more likely to occur in those with milder disease but less likely to occur in those ever on biologics. Overall, OV therapy appeared to be well-tolerated in the literature with no reports of vancomycin-resistant enterococci (VRE) or other significant adverse events.

The use of antibiotics to treat patients with IBD has been previously reported by a meta-analysis,^22^ however, more recent systematic reviews have questioned the effectiveness of antibiotics.^23,24^ Furthermore, OV has been shown to be clinically effective as an adjuvant therapy even in IBD patients without PSC.^47–49^ The pathophysiological mechanism underlying the proposed beneficial effects of OV in PSC-IBD patients is speculatory at this time, however, it likely includes immunomodulation, bile acid regulation, and microbiome manipulation. Abarbanel et al^26^ illustrated that OV increases levels of anti-inflammatory transforming growth factor-beta (TGF-β) as well as regulatory T cells in 14 PSC-IBD children. Oral vancomycin may also decrease the production of TNF-α which has been implicated in T-cell reactivity in PSC patients.^50,51^ Vaughn et al^28^ have demonstrated that OV in PSC-IBD patients decreases the production of secondary bile acids and lastly, OV results in fecal microbiome changes that coincide with clinical and biochemical improvement and result in a decrease in *Fusobacterium* and *Haemophilus* as well as an increase in *Veillonella.*^42^

This systematic review has several strengths. We used a thorough search strategy to ensure that all relevant studies were included. Our systematic review helps identify knowledge gaps in OV and PSC-IBD, which suffer from a dearth of robust prospective data looking at primary IBD outcomes. The 2 cohort studies and one RCT captured IBD activity as a secondary outcome using either the sole descriptor of diarrhea or the PGA. Though Ricciuto et al^40^ assessed the PGA of IBD clinical activity as a primary outcome, future studies would benefit from the use of standardized symptom indices such as the partial Mayo score or pediatric ulcerative colitis activity index, protocolized objective disease monitoring in the form of FCP and endoscopy, consistent OV dosing and homogenous follow-up.^52^ There is probably some benefit with OV, however, further mechanistic studies are also warranted such as the PSC-Vanc study, which Quraishi and colleagues from the University of Birmingham have completed recruitment for (NCT05376228).

Despite the limitations of the current literature, the existing data is encouraging. Oral vancomycin appears safe and effective in at least a subset of PSC-IBD patients. In a disease where up to 1/3 of PSC-IBD patients post-orthotopic liver transplantation may experience flares despite ongoing immunosuppression^3^ and with IBD being a risk factor for PSC recurrence and graft failure,^7^ OV is an attractive option and avoids the morbidity related to colectomy including the risk of chronic pouchitis.^53^ The authors propose that OV, as an off-label use, could be considered as an alternative therapy in PSC-IBD patients having failed at least 3 advanced therapies or possibly as an adjuvant therapy in patients who develop secondary loss of response to any advanced therapy. Further research into therapies for PSC-IBD will also provide clues into the pathogenesis of PSC-IBD—specifically, the interplay of the liver-gut microbiome axis. Although Shah et al^45^ and Tan et al^32^ revealed that none of the PSC-IBD patients treated with OV had evidence of VRE, this remains a concern, especially in PSC-IBD patients who can develop recurrent cholangitis. Ultimately, a well-powered RCT is needed to better assess the effectiveness, safety, and durability of OV therapy. Until then, the use of OV in the PSC-IBD patient should be used with cautious optimism.

## Supplementary Data

Supplementary data is available at *Inflammatory Bowel Diseases* online.

izae257_suppl_Supplementary_Material

## Data Availability

All data are incorporated into the article and its Supplementary Material.
