# Supplementary material for: The Effectiveness of Oral Vancomycin on Inflammatory Bowel Disease in Patients With Primary Sclerosing Cholangitis: A Systematic Review
Source: Inflamm Bowel Dis. 2024 Nov 4;31(7):2027–35. doi: 10.1093/ibd/izae257 (PMC12235138; doi:10.1093/ibd/izae257)
Supplement: izae257_suppl_Supplementary_Material [file izae257_suppl_supplementary_material.docx]

**Supplemental Data**

| **Supplementary Table 1: Quality Assessment of Case Reports and Series** | | | | | | | | | | | | | | | | |
| --- | --- | --- | --- | --- | --- | --- | --- | --- | --- | --- | --- | --- | --- | --- | --- | --- |
|  | **^8^** | **^9^** | **^12^** | **^26^** | **^37^** | **^31^** | **^42^** | **^33^** | **^43^** | **^30^** | **^44^** | **^32^** | **^29^** | **^45^** | **^41^** | **^46^** |
| **Selection** | 0 | 0 | 1 | 0 | 0 | 0 | 0 | 0 | 0 | 0 | 0 | 0 | 1 | 1 | 0 | 1 |
| **Ascertainment** | 1 | 1 | 1 | 1 | 1 | 1 | 1 | 1 | 1 | 1 | 1 | 1 | 1 | 1 | 1 | 1 |
|  | 1 | 1 | 1 | 1 | 1 | 1 | 1 | 1 | 1 | 1 | 1 | 1 | 1 | 1 | 1 | 1 |
| **Causality** | 1 | 1 | 1 | 1 | 0 | 1 | 1 | 1 | 1 | 1 | 1 | 1 | 1 | 1 | 1 | 1 |
|  | 0 | 1 | 0 | 1 | 0 | 0 | 0 | 0 | 0 | 1 | 0 | 0 | 0 | 0 | 1 | 1 |
|  | 0 | 0 | 1 | 0 | 1 | 0 | 0 | 1 | 0 | 0 | 1 | 0 | 0 | 0 | 0 | 0 |
|  | 1 | 1 | 1 | 1 | 0 | 1 | 1 | 1 | 0 | 1 | 1 | 1 | 1 | 1 | 1 | 1 |
| **Reporting** | 1 | 1 | 1 | 1 | 1 | 1 | 1 | 1 | 1 | 1 | 1 | 1 | 1 | 1 | 1 | 1 |
| **Total Score** | 5 | 6 | 7 | 6 | 4 | 5 | 5 | 6 | 4 | 6 | 6 | 5 | 6 | 6 | 6 | 7 |

| **Supplementary Table 2: Cochrane Collaboration Risk of Bias Tool for Quality Assessment of the Clinical Trial** | |
| --- | --- |
| **Selection** | **Rahimpour et al., 2016** ^38^ |
| Random sequence generation | Low risk of bias |
| Allocation concealment | Low risk of bias |
| **Performance** |  |
| Blinding (participants and personnel) | Low risk |
| **Detection** |  |
| Blinding (outcome assessment) | Low risk |
| **Attrition** |  |
| Incomplete outcome data | Low risk (1 participant dropped from each group) |
| **Reporting** |  |
| Selective reporting | Low risk of bias |
| **Other** |  |
| Other sources of bias | Unequal distribution between the experimental and control groups |

| **Supplementary Table 3: Newcastle-Ottawa Quality Assessment of Cohort Studies** | | | |
| --- | --- | --- | --- |
| **Selection** | **Deneau et al., 2021** ^1^ | **Ali et al., 2020** ^39^ | **Ricciuto et al., 2024** ^40^ |
| Representativeness of the exposed cohort | Truly representative (1) | Truly representative (1) | Truly representative (1) |
| Selection of the non-exposed cohort | Same community (1) | N/A (open label) | Same community (1) |
| Ascertainment of exposure | Secure records (1) | Secure records (1) | Secure records (1) |
| Demonstration that outcome of interest was not present at start of study | No (0) | Yes (1) | No (0) |
| **Comparability** |  |  |  |
| Study controls for age and sex | No (0) | No (0) | Yes (1) |
| Study controls for any additional factor: | Yes (1): liver biochemistry, PSC disease phenotype, concomitant medications | No (0) | Yes (1): IBD type, PGA at IBD diagnosis, previous and current IBD medical therapy |
| **Outcome** |  |  |  |
| Assessment of outcome | Record linkage (1) | Record linkage (1) | Record linkage (1) |
| Was follow-up long enough for outcomes to occur | Yes (1): minimum 3 months, up to a year.  * 4 UDCA patients stopped therapy at 2 months | Yes (1): minimum 3 months, up to varying years. | Yes (1): minimum 3 months, median 2.5 years |
| Adequacy of follow up of cohorts | All followed up (1) | All followed up (1) | N/A (0) |
| **Total Score** | **Good quality:** 3 in selection domain AND 1 in the comparability domain AND 3 in the outcome domain | **Poor quality:** 3 in selection domain AND 0 in the comparability domain AND 3 in the outcome domain | **Good quality:** 3 in selection domain AND 2 in the comparability domain AND 2 in the outcome domain |

**Search strategy**

**Ovid MEDLINE(R) ALL <1946 to March 21, 2024>**

**Searched March 22, 2024**

1 (inflammatory bowel disease* or IBD or ulcerative colitis or Crohn* disease).mp. 140109

2 inflammatory bowel diseases/ or colitis, ulcerative/ or crohn disease/ 100201

3 1 or 2 144712

4 (cholangitis or PSC or autoimmune liver disease).mp. 29426

5 Cholangitis, Sclerosing/ 4783

6 4 or 5 29426

7 anti-infective agents/ or anti-bacterial agents/ or amoxicillin-potassium clavulanate combination/ or ciprofloxacin/ or fidaxomicin/ or metronidazole/ or rifaximin/ or vancomycin/ 484686

8 (ciprofloxacin or metronidazole or rifaximin or vancomycin or amoxicillin-clavulanic acid or anti?biotic* or anti?microbial* or anti?bacterial*).mp. 752542

9 7 or 8 893686

10 3 and 6 and 9 135

**Embase <1974 to 2024 March 21>  (OVID interface)**

**Searched March 22, 2024**

1 (inflammatory bowel disease* or IBD or ulcerative colitis or Crohn* disease).tw,kf. 214143

2 (cholangitis or PSC or autoimmune liver disease).tw,kf. 43712

3 antibiotic agent/ or ciprofloxacin/ or metronidazole/ or rifaximin/ 555259

4 polypeptide antibiotic agent/ or vancomycin/ 138785

5 antiinfective agent/ 219039

6 exp amoxicillin plus clavulanic acid/ 51812

7 fidaxomicin/ 2058

8 (ciprofloxacin or metronidazole or rifaximin or vancomycin or amoxicillin-clavulanic acid or anti?biotic* or anti?microbial* or anti?bacterial*).mp. 1318084

9 3 or 4 or 5 or 6 or 7 or 8 1390861

10 1 and 2 and 9 436

**CINAHL Plus with Full Text (Ebscohost interface)**

Date searched: March 22, 2024

Results: 15

S1   (MH "Inflammatory Bowel Diseases") OR (MH "Colitis, Ulcerative") OR (MH "Crohn Disease") or "inflammatory bowel disease*" or IBD or "ulcerative colitis" or "Crohn* disease"

S2  (MH "Cholangitis, Sclerosing") or cholangitis or PSC or "autoimmune liver disease"

S3  (MH "Fidaxomicin") OR (MH "Vancomycin") OR (MH "Rifaximin") OR (MH "Antibiotics") OR (MH "Antiinfective Agents") OR (MH "Metronidazole") OR (MH "Ciprofloxacin") OR ciprofloxacin or metronidazole or rifaximin or vancomycin or amoxicillin-clavulanic acid or antibiotic* or antimicrobial* or antibacterial* or anti-biotic* or anti-microbial* or anti-bacterial*

S4 S1 AND S2 AND S3

**Scopus (advanced search)**

Date searched: March 22, 2024

Results: 514

TITLE-ABS-KEY( "inflammatory bowel disease*" or IBD or "ulcerative colitis" or "Crohn* disease") AND TITLE-ABS-KEY(cholangitis or PSC or "autoimmune liver disease") AND TITLE-ABS-KEY(ciprofloxacin or metronidazole or rifaximin or vancomycin or "amoxicillin-clavulanic acid" or antibiotic* or anti-biotic* or antimicrobial* or anti-microbial* or antibacterial* or anti-bacterial*)

**Web of Science Core Collection (A&HCI , ESCI , CPCI-SSH , CPCI-S , SCI-EXPANDED , SSCI)**

Date searched: March 22, 2024

Results: 181

TS=( "inflammatory bowel disease*" or IBD or "ulcerative colitis" or "Crohn* disease") AND TS=(cholangitis or PSC or "autoimmune liver disease") AND TS=(ciprofloxacin or metronidazole or rifaximin or vancomycin or "amoxicillin-clavulanic acid" or antibiotic* or anti-biotic* or antimicrobial* or anti-microbial* or antibacterial* or anti-bacterial*)
